# Microsurgical education for medical students: systematic review

**DOI:** 10.1590/acb412726

**Published:** 2026-06-29

**Authors:** Michelly Moreira Campos, Ana Cristina Aoun Tannuri, Edna Frasson de Souza Montero, Eric Toshiyuki Nakamura, Giovanna Mattos Ferreira, Pedro Carvalho Cassino, Cinthia Lanchotte, Lucas Albuquerque Chinelatto, Filipe Miranda de Oliveira Silva, Flavio Henrique Ferreira Galvão

**Affiliations:** 1 Universidade de São Paulo – Faculdade de Medicina – Departamento de Gastroenterologia – São Paulo (SP) – Brazil.; 2 Universidade de São Paulo – Faculdade de Medicina – Laboratório de Investigação Médica – São Paulo (SP) – Brazil.

**Keywords:** Microsurgery, Models, Theoretical, Rats, Education, Medical

## Abstract

**Purpose:**

To perform a systematic review about microsurgical training for medical students.

**Methods:**

A systematic search was conducted in several databases. Screening was performed by two independent reviewers based on predetermined criteria, following PRISMA 2020 guidelines.

**Results:**

A total of 433 articles were obtained. After screening, 40 were included. Teaching methods were heterogeneous and restricted to a few countries. There are specific methods to assess microsurgery training, general surgical activities, learning curve and anxiety. The most used materials were surgical gloves, silicone tubes, chicken thighs and rats. Animals were not essential in early stages of education. There was a variation between one and 24 sessions regarding teaching frequency and between 3 and 120 hours of teaching period. The average age of students was 23.7, and 67.5% were men. Teaching groups had an average of 5.8 students per group. Physical activity and anxiety had negative effect on microsurgical ability gain, while caffeine had positive effect. Alcohol and sleep deprivation had no significant effect.

**Conclusion:**

Students with adequate training could achieve performance similar to that obtained by experts. Microsurgical education is realistic during medical graduation and may enable better career choices. More investigations are needed to improve this training and expand knowledge to other medical schools.

## Introduction

Microsurgery emerged in the 20th century and nowadays represents the pinnacle of medical precision^1^. In clinical practice, microsurgery plays a fundamental role expanding treatments for complex diseases involving neurosurgery^2^, plastic surgery^3^, hand surgery^4^, ophthalmology^5^, among others. Another critical application of this procedure is translational scientific research using small animals such as rodents, which fosters the development of innovative therapies^6^.

The teaching of surgery is a highly complex process involving difficult evaluation methods of skill acquisition. It often requires extensive training, sophisticated instruments and expensive surgical sutures. Research on microsurgical education helps allocate tasks based on skills, reduces future losses from inability, monitors the training costs and assesses student ability gain and learning curves^7^. Inadequate training is associated with increased surgical complications and high treatment costs. Therefore, surgical education is a public health issue^8^.

Due to its complexity, microsurgical training typically occurs at later stages of surgical carrier, with scant opportunities for medical students^9^. However, its increasing importance prospects that microsurgery will be gradually integrated into medical education following the same path as regular surgery in the past^10^.

Nevertheless, there is a lack of review articles assessing microsurgical training for medical students. Thus, the objective of this manuscript was to perform a systematic review about method of microsurgical training for medical students.

## Methods

This systematic review was conducted according to PRISMA 2020 guidelines and registered in the PROSPERO system, with the identification number CRD420251169243. The data search was performed by evaluating the PubMed, Virtual Health Library (BVS), Embase, Scopus, and Web of Science databases, using the following terms: “(teaching OR education) AND (microsurgery) AND (medical students)”, without filters or period limits.

### Eligibility criteria

The inclusion criteria were all articles that evaluate the teaching of microsurgery applied to medical students. Exclusion criteria include specialist-focused training, absence of microsurgery training, absence of medical students in the training, and lack of reference to the educational process.

### Data selection

From the articles found, the selection was carried out by two independent reviewers, who compared the results, jointly resolving any discrepancies with the aid of the Rayyan program, using title and abstract analysis.

### Data collection

The complete reading of the articles was carried out by the same two reviewers. They also worked independently in this phase, collecting specific data from the articles on predefined topics and highlighting other relevant information. A study design table was created for greater transparency and to make it easier to find the original articles (Table 1). The predefined analysis points included materials used, teaching methodology, number of students per group, duration of teaching, sociodemographic information, influence of non-surgical factors on the learning curve, training evaluation methods, and comparison between medical students, residents, and groups of experienced surgeons. From each study, all results compatible with each expected outcome domain were collected.

**Table 1:** Study design.

**Study**	**Country**	**Type of study**	**Participants**	**Study design**	**Result**
Mikó et al.^11^	Hungary	Descriptive methodological study	Medical students, researchers, doctors and specialists	Furka microsurgery course, with 20 hours distributed across five 4-hour classes	15 years of experience demonstrated that the method allows for progressive and structured learning of microsurgery, highlighting the effectiveness of personalized follow-up
Müller et al.^12^	Germany	Cross-sectional observational study	64 medical students	Evaluation of a practical (wet lab) course in microsurgical suturing in ophthalmology with subsequent assessment by questionnaire	There were an increased interest in ophthalmology after the course and strong agreement regarding the desire for more microscopic training
Onoda et al.^13^	Japan	Descriptive observational study	29 medical students	Review of the results of the microsurgery training program	There was a negative correlation between the number of trials in non-animal stages and the number of rats used afterward, suggesting that training first in non-living models reduces the use of animals and allows for effective learning of microsurgery
Liu et al.^14^	Canada	Descriptive methodological study	Six medical students, eight residents, and 10 experienced doctors	Multilevel course in ophthalmic microsurgery in a wet lab, with participant feedback collection	The organization, course structure, and positive participant feedback are described, highlighting the viability of multilevel and collaborative programs for surgical training
Deuchler et al.^15^	Germany	Prospective observational study	79 medical students	Students underwent training in an ophthalmic microsurgery simulator and were evaluated using objective simulator scores and subjective questionnaires before and after training	The training significantly increased the students' self-perception of microsurgical skills, although there was no association between simulator performance and interest in the specialty
Cole et al.^16^	United States of America	Uncontrolled trial	20 medical students	Students participated in a class and performed a wet lab on corneal suturing. Pre- and post-intervention questionnaires	There was a significant increase in comfort with various microsurgical skills, interest in ophthalmology increased, and intrinsic motivation was high
Bastos and Silva^17^	Brazil	Descriptive methodological study	Medical students	Development of a synthetic model for basic surgical training made of ethylene-vinyl-acetate plates	The synthetic model has been described as a simple, reproducible, and low-cost alternative for the initial teaching of basic surgical skills to medical students before clinical practice
Kinshoku et al.^18^	Brazil	Descriptive methodological study	Medical students	Basic microsurgery training model using Wistar rat cadavers from discarded specimens from other experiments, allowing for microanastomoses	The experimental model enabled safe, low-cost, and ethical microsurgical training
Lin et al.^19^	United Kingdom	Descriptive observational study	42 medical students	Practical microsurgical course with specialized supervision. Evaluation conducted through qualitative questionnaires before and after the workshop	There has been progress and increased confidence in microsutures. In total 67٪ considered it important for their future career. Feedback indicated a need for more time and better equipment
Jensen et al.^20^	United States of America	Longitudinal observational study	12 medical students	Development of low-cost, reusable microsurgery kits using surplus surgical materials. Students performed suturing/anastomosis, evaluated using a performance scale at two time points with a six-week interval	There was an improvement in performance scores and a reduction in time spent on suturing and anastomosis tasks after six weeks, suggesting that the kits are useful educational tools for early introduction to microsurgery
Scholz et al.^21^	Germany	Descriptive observational study	36 medical students	Implementation and evaluation of a microsurgery training program without the use of animal models	The training produced good to excellent results among the students. The animal-free program proved viable for initial microsurgery training and for comparative skill assessment
Pavlidis et al.^22^	United States of America	Longitudinal observational study	15 medical students	Informal microsurgical training was conducted weekly for five weeks, with performance analysis by two independent reviewers, including anxiety, workload and sympathetic activation	Sympathetic activation and anxiety levels did not correlate with performance or speed. Training in an informal setting may facilitate skill acquisition by reducing high stress levels
Maluf Junior et al.^23^	Brazil	Descriptive methodological study	Medical students	Microsurgical training using pig spleens discarded after splenectomy	The model proved effective for initial learning of vascular microsurgery, at low cost and without the need to sacrifice new animals
Sakamoto et al.^24^	Japan	Randomized controlled trial	86 medical students	Students were randomized to either hands-on training on a benchtop simulator or video-based learning, followed by 40 minutes of microsuturing practice and subsequent evaluation	The simulator group completed the task in less time than the video group. The final suture quality was higher in the simulator group, but without a significant difference. Practical training on a simulator proved more effective for learning microsuture
Froschauer et al.^25^	Austria	Randomized experimental study	15 medical students and 15 experienced microsurgeons	Each participant performed two end-to-end anastomoses on a chicken thigh model, one with and one without music	Music had no significant overall effect on run time or performance. However, among experienced surgeons, preferring to work with music was associated with better scores on the Stanford Microsurgery and Resident Training (SMaRT) scale
Al Omran et al.^26^	United Kingdom	Analytical observational study	40 medical students and six residents	Participants performed consecutive end-to-end microvascular anastomoses; performance was assessed by hand movement analysis and correlated with habitual levels of physical activity	Higher levels of physical activity correlated with worse microsurgical performance among beginners
Sudario-Lumague et al.^27^	Taiwan	Analytical observational study	578 medical students	Students underwent basic microsurgery training and were evaluated by a senior consultant. Scores between men and women were statistically compared	There was no significant difference in performance between male and female students, indicating similar microsurgical skills between the groups
Hanrahan et al.^28^	United Kingdom	Analytical observational study	40 medical students	Students performed dural suturing on an ex-vivo porcine model during a neurosurgery course, with evaluations of performance, dexterity, anxiety, and tremor	Physiological tremor was not significantly associated with microsurgical dexterity. Higher subjective perception of anxiety correlated with worse performance
Mücke et al.^29^	Germany	Prospective comparative study	59 medical students and 19 surgeons	Intensive 14-day microsurgery course, with practical and theoretical evaluation by two independent and blinded examiners for comparison between students and surgeons	Students obtained significantly higher scores than surgeons in practical (13.71 versus 11.73; p < 0.0001) and theoretical (15.27 versus 13.50; p = 0.009) exams and greater course attendance
Teo et al.^30^	Singapore	Randomized controlled trial	42 medical students (24 in the mass-learning group; 18 in the spaced-learning group)	Students were randomized to either single 8-hour session microsuture training (mass-learning) or weekly 2-hour sessions for four weeks (spaced-learning), with an assessment conducted one month after training	The spaced learning group showed significantly higher scores in suture accuracy after one month, indicating better skill retention
Almeland et al.^31^	Norway	Randomized controlled trial	46 medical students randomized into two groups	The intervention group received training in macro and microsurgery; the control group received only microsurgery training. After training, all participants performed macrosurgical suturing tasks and were evaluated	The intervention group showed worse macrosurgical performance
Jensen et al.^32^	United States of America	Randomized controlled trial	24 medical students (12 in the LazyBox simulator group; 12 in the control group)	Students undergo longitudinal practice on a low-fidelity microsurgery simulator (LazyBox) (group 1) or no additional practice (group 2). Assessments are performed before and six weeks after the intervention	Both groups showed improvement in the tasks, with no statistically significant differences. The use of the LazyBox simulator did not demonstrate any significant additional benefit
Nemeth et al.^33^	Hungary	Descriptive study	470 residents e medical students	Summary of 50 years of experience in microsurgical education, with emphasis on individualized training, standardization, skills assessment, and structured feedback	Experience showed that early and progressive exposure improves performance in subsequent residencies. The importance of adequate duration, individualized training and objective assessment is highlighted
Galvão et al.^34^	Brazil	Prospective observational study	38 medical students	Description of a microsurgical training course in rat intestinal transplantation	Low completion rate attributed to lack of time, patience, frustration, or technical difficulty. Medical students showed a low completion rate in complex microsurgical models
Zhang et al.^35^	United States of America	Descriptive observational study	Medical students	Review of institutional experience in creating microsurgery research programs for medical students	Microsurgery research programs for students provided high-quality technical training, contributed to scientific output and influenced career choices in surgical fields
Climov et al.^36^	Romania	Comparative experimental study	One medical student and one microsurgeon	Comparison of the performance between a student and an experienced microsurgeon in performing hemifacial transplantation in rats, analyzing the learning curve	The student showed a progressive reduction in operating time and ischemia, approaching the performance of a specialist after nine transplants
Beier et al.^37^	Germany	Descriptive observational study	44 medical students	Implementation of an extracurricular microsurgery course for medical students with training in end-to-end arterial anastomosis in chicken thigh, with evaluation conducted through questionnaires before and after the course	The course led to a significant improvement in self-assessment of microsurgical skills, a high level of participant satisfaction, and 82٪ reported a positive influence on their future choice of specialty
Couceiro et al.^38^	United States of America	Experience report	Medical students	Description of a continuous microsurgery training regimen using non-living animal models (chicken thighs and wings)	The training regimen using non-living tissues has proven to be accessible, economical, useful for developing microsurgical skills, and avoids the use of live animals in training
Bhandarkar et al.^39^	United States of America	Pilot feasibility study	Medical students	Reproduction and implementation of a low-cost microsurgical training system (smartphone for magnification + "Lazy Glasses") for performing tasks	The simulation system was easy to assemble, low cost (< $ 50) and viable for microsurgical training in medical students, with the potential to expand access to microsurgical training
Haran et al.^40^	New Zealand	Prospective observational study	Nine medical students and 11 residents	Four-hour dry lab course for training in basic microsurgical skills. Subjective assessment of skills before and after the course using a Likert scale	There was a significant improvement in self-assessment of microsurgical skills. Early exposure increases confidence and perceived ability
Zyluk et al.^41^	Poland	Experimental study	10 medical students	Students performed a microsuture test (“six-stitches test”) on a latex glove before and after exposure to three conditions: caffeine consumption, ingestion of a small dose of alcohol, and physical exercise	Caffeine had a significant positive effect on performance. Physical exercise performed immediately before the task had a negative effect. A small dose of alcohol had a minimal effect on performance
Micko et al.^42^	Austria	Experimental study	10 medical students and 10 neurosurgery residents	Each participant performed a microsurgical task in the NeuroTouch virtual reality simulator under two conditions: well-rested (baseline) and after a sleep interruption at night (stress test)	After sleep interruption, the total score improved significantly (45.1 → 48.7; p = 0.048), the execution time remained stable, and excessive force varied by hand, but self-assessment worsened
Fulton et al.^43^	United States of America	Case study	One medical student	Individual microsurgical training with knot tying and anastomosis practice on synthetic vessels, under the supervision of an experienced instructor	The participant achieved a technical level equivalent to a third-year surgical resident. Early exposure can develop adequate microsurgical skills
Kaplan et al.^44^	United States of America	Randomized trial	10 medical students and 10 residents	Randomization into two groups: control (endoscopic dissection on a silicone anatomical specimen) and intervention (prior training in microvascular dissection on a chicken wing model). Single-blind assessment of time to complete the task and dissection quality	Prior microsurgical training significantly improved the time and quality of dissection in all groups, with the greatest benefit observed in medical students
Neudert et al.^45^	Germany	Randomized trial	30 medical students and six otosurgeons	Group 2 underwent ossicular and tympanic membrane reconstruction on days 1, 7, 14, and 21; group 1 underwent reconstruction on days 1 and 21 and only observed on days 7 and 14. The results were evaluated in comparison with otolaryngologists	The group with more frequent training showed significant improvement, but it did not reach the specialists' level. The training increased the students' interest in the surgical specialty
Ilgner et al.^46^	Germany	Descriptive observational study	Medical students	Implementation of training in ontological microsurgery using a plastic ear model and a high-definition stereoscopic video system coupled to a surgical microscope	Students improved their visuomotor coordination and gained greater confidence in their manual skills. The system facilitated group feedback and sparked interest in microsurgical specialties
Furka et al.^47^	Hungary	Retrospective observational study	263 medical students	Training courses in suturing/microsurgery, with initial and final evaluation of time, safety, knot stability, and suture thread integrity	There was a significant reduction in knot tying time after training. Individualized training is more suitable than mass training for teaching microsurgery
von Reibnitz et al.^48^	Switzerland	Preclinical prospective trial	13 participants: medical students, residents, and attending physicians	In three training sessions, each participant performed nine microvascular anastomoses using a robotic system. Videos of the anastomoses were blindly evaluated by a specialist	There were a significant reduction in anastomosis time and an improvement in scores throughout the sessions. Prior surgical experience initially correlated with better performance, but this difference disappeared in the last session
Sakamoto et al.^49^	Japan	Non-randomized controlled experimental study	25 medical students and nine neurosurgeons	Students trained in microanastomosis for eight weeks. One group received weekly feedback from a specialist via video call, and the other group practiced self-study using DVDs, with a final practical exam blindly evaluated by two specialists; performance was also compared with neurosurgeons	The group that received expert feedback achieved significantly higher scores in anastomosis than the self-learning group. This group also scored higher than the neurosurgeons

Source: Elaborated by the authors.

### Risk of bias assessment and data synthesis

Risk of bias assessment was performed independently by two reviewers using the Critical Appraisal Skills Program (CASP) tool for qualitative studies (Table 2). Disagreements were resolved by consensus. Qualitative data were presented descriptively, and quantitative analyses were expressed as simple and weighted averages.

**Table 2:** Risk of bias analysis.

Study	Clear aim	Appropriate methodology	Aims addressed by the study design	Adequate recruitment	Reliable data collection	Bias controlled	Ethical consideration	Accurate data analysis	Clear statement of findings	Relevance	Overall risk
Mikó et al.^11^	Yes	Yes	Yes	Yes	Yes	Yes	Yes	Yes	Yes	Yes	Low
Müller et al.^12^	Yes	Yes	Yes	Yes	Yes	Yes	Yes	Yes	Yes	Yes	Low
Onoda et al.^13^	Partial	Yes	Yes	Yes	Yes	Yes	Yes	Yes	Yes	Yes	Low
Liu et al.^14^	Yes	Yes	Yes	Yes	Yes	Yes	Yes	Yes	Yes	Yes	Low
Deuchler et al.^15^	Yes	Yes	Yes	Yes	Yes	Yes	Yes	Yes	Yes	Yes	Low
Cole et al.^16^	Yes	Yes	Yes	Yes	Yes	Yes	Yes	Yes	Yes	Yes	Low
Bastos and Silva^17^	Yes	Yes	Yes	Can't tell	Yes	Yes	Yes	Yes	Yes	Yes	Low
Kinshoku et al.^18^	Yes	Yes	Yes	Can't tell	Yes	Yes	Yes	Yes	Yes	Yes	Low
Lin et al.^19^	Yes	Yes	Yes	Yes	Yes	Yes	Yes	Yes	Yes	Yes	Low
Jensen et al.^20^	Yes	Yes	Yes	Yes	Yes	Yes	Yes	Yes	Yes	Yes	Low
Scholz et al.^21^	Yes	Yes	Yes	Yes	Yes	Partial	Yes	Yes	Yes	Yes	Low
Pavlidis et al.^22^	Yes	Yes	Yes	Yes	Yes	Yes	Yes	Yes	Yes	Partial	Low
Maluf Junior et al.^23^	Yes	Yes	Yes	Yes	Yes	Yes	Yes	Yes	Yes	Yes	Low
Sakamoto et al.^24^	Yes	Yes	Yes	Yes	Yes	Yes	Yes	Yes	Yes	Yes	Low
Froschauer et al.^25^	Yes	Yes	Yes	Yes	Yes	Yes	Yes	Yes	Yes	Yes	Low
Al Omran et al.^26^	Yes	Yes	Yes	Yes	Yes	Can't tell	Yes	Yes	Yes	Yes	Low
Sudario-Lumague et al.^27^	Yes	Yes	Yes	Yes	Yes	Yes	Yes	Yes	Yes	Yes	Low
Hanrahan et al.^28^	Yes	Yes	Yes	Yes	Yes	Yes	Yes	Yes	Yes	Yes	Low
Mücke et al.^29^	Yes	Yes	Yes	Yes	Yes	Yes	Yes	Yes	Yes	Yes	Low
Teo et al.^30^	Yes	Yes	Yes	Yes	Yes	Yes	Yes	Yes	Yes	Yes	Low
Almeland et al.^31^	Yes	Yes	Yes	Yes	Yes	Yes	Yes	Yes	Yes	Partial	Low
Jensen et al.^32^	Yes	Yes	Yes	Yes	Yes	Yes	Yes	Yes	Yes	Yes	Low
Nemeth et al.^33^	Partial	Yes	Yes	Yes	Partial	Yes	Yes	Yes	Yes	Yes	Moderate
Galvão et al.^34^	Yes	Yes	Yes	Yes	Yes	Yes	Yes	Yes	Yes	Yes	Low
Zhang et al.^35^	Yes	Yes	Yes	Yes	Yes	Yes	Yes	Yes	Yes	Yes	Low
Climov et al.^36^	Yes	Yes	Yes	Yes	Yes	Yes	Yes	Yes	Yes	Yes	Low
Beier et al.^37^	Yes	Yes	Yes	Yes	Yes	Can't tell	Yes	Yes	Yes	Yes	Low
Couceiro et al.^38^	Yes	Yes	Yes	Yes	Partial	Yes	Yes	Yes	Yes	Yes	Low
Bhandarkar et al.^39^	Yes	Yes	Yes	Yes	Yes	Yes	Yes	Yes	Yes	Yes	Low
Haran et al.^40^	Yes	Yes	Yes	Yes	Yes	Yes	Yes	Partial	Partial	Yes	Moderate
Zyluk et al.^41^	Yes	Yes	Yes	Yes	Yes	Yes	Yes	Yes	Yes	Yes	Low
Micko et al.^42^	Yes	Yes	Yes	Yes	Yes	Yes	Yes	Yes	Yes	Partial	Low
Fulton et al.^43^	Yes	Yes	Yes	Yes	Yes	Yes	Yes	Yes	Yes	Yes	Low
Kaplan et al.^44^	Yes	Yes	Yes	Yes	Yes	Yes	Yes	Yes	Yes	Yes	Low
Neudert et al.^45^	Yes	Yes	Yes	Yes	Yes	Yes	Yes	Yes	Yes	Yes	Low
Ilgner et al.^46^	Yes	Yes	Yes	Yes	Partial	Yes	Yes	Partial	Partial	Yes	Moderate
Furka et al.^47^	Yes	Yes	Yes	Yes	Yes	Yes	Yes	Yes	Yes	Yes	Low
von Reibnitz et al.^48^	Yes	Yes	Yes	Yes	Yes	Yes	Yes	Partial	Partial	Yes	Moderate
Sakamoto et al.^49^	Yes	Yes	Yes	Yes	Yes	Yes	Yes	Yes	Yes	Yes	Low

Source: Elaborated by the authors.

## Results

**Figure 1:**
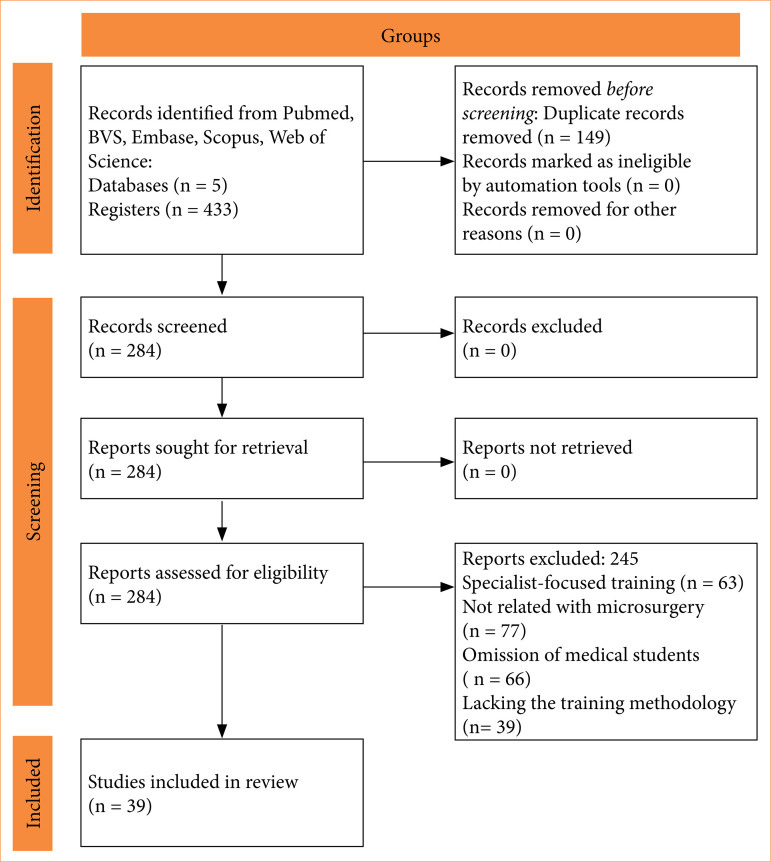
Diagram of the literature’s review.

Source: Elaborated by the authors.

A total of 433 articles were assessed, 114 from PubMed, 27 from BVS, 140 from Embase, 75 from Scopus, and 77 from Web of Science, with the last consultation on November 22nd, 2024. A total of 149 duplicates were excluded, lasting 284 articles. From these 284 articles, 244 were excluded, 63 due to specialist-focused training, 77 not related with microsurgery topic, 65 for omitting medical students and 39 for lacking the methodology of the training. In the end, 40 articles were included in this survey (Fig. 1).

Most of the articles present microsurgery as elective courses/subjects^11–20^ or as part of research projects^21–32^. In one article, the institution included microsurgical training as a mandatory subject^33^.

**Figure 2:**
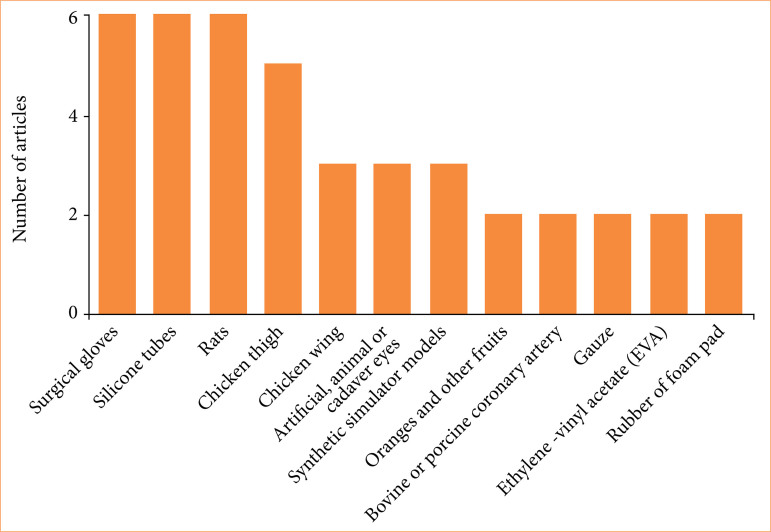
Materials used to teach microsurgery to medical students and the number of times each was described in the articles.

Source: Elaborated by the authors.

Various materials were employed for the training, with emphasis on the surgical glove^21,22,27,29,33,34^, silicone tubes^13,20,24,31,32,35^, rats^11,13,18,34–36^ and chicken thighs^25,29,33,37,38^ (Fig. 2). Other materials less used included pig skin^12^, newspaper^33^, goat or canine sciatic nerve^21^, pig spleen^23^, and pig dura mater^28^.

Kinshoku et al.^18^ advocates for the use of already deceased rats for medical student training, with the cadavers originating from prior practices in other health-related courses and researches. This procedure allows the rational use of animals and provides training closer to real-life scenarios.

Galvão et al.^34^ introduced a training of intestinal transplantation technique in rats for medical students and observed that most students abandoned the training before completion of the investigation. This result was attributed to the high complexity of the procedure, lack of time, and frustration. Only 5% of the students were able to complete the total procedure of intestinal transplantation technique in the end of the training.

Two articles^20,39^ mention the use of smartphone magnification in locations with limited access to bench microscopes. However, its application for medical students^32^ had poor results, which was explained by the failure to convert the learned skills to the operating microscope.

**Figure 3:**
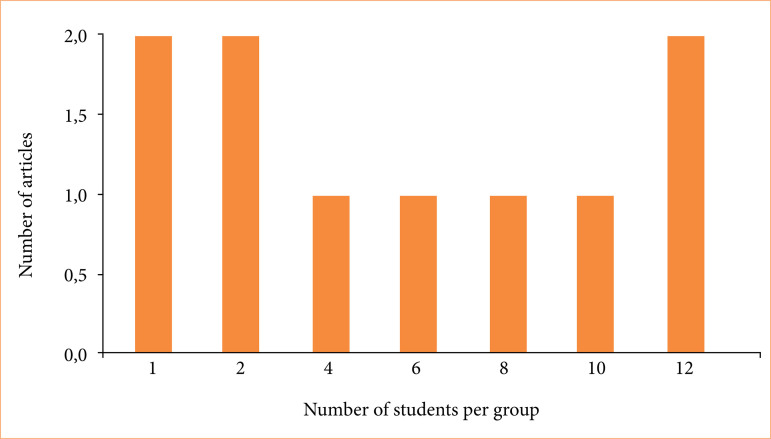
Number of students per teaching group in microsurgery and the number of times it was applied.

Source: Elaborated by the authors.

The number of students per teaching group was recorded (Fig. 3). Beier et al.^37^ highlight “peer-assisted learning” with excellent results. In this review, the average was 5.8 students per teaching group.

**Figure 4:**
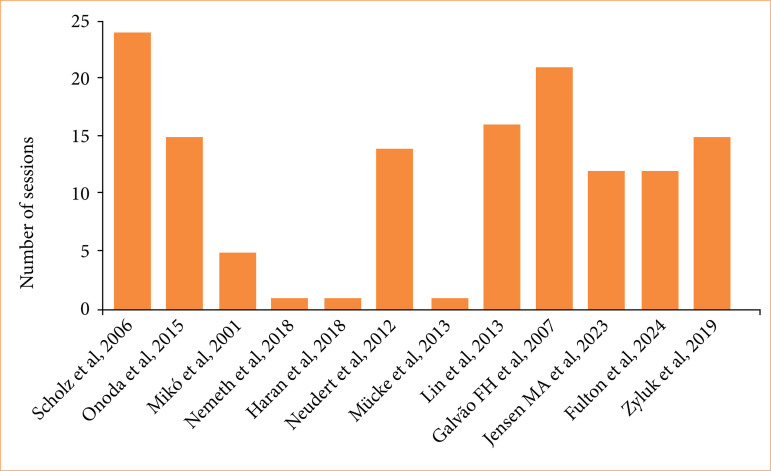
Total number of microsurgery training sessions for medical students in each article.

Source: Elaborated by the authors.

**Figure 5:**
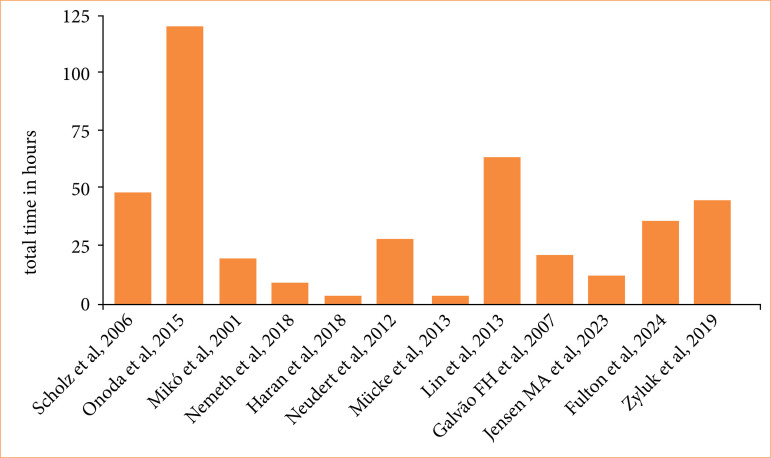
Microsurgery training time spent on each article.

Source: Elaborated by the authors.

Regarding teaching time, information on the number of sessions (Fig. 4) and total training time in hours (Fig. 5) was recorded.

Regarding skill retention, one study30 compared suture accuracy after course of 8 hours in a single day with other group that had four sessions of 2 hours each for one month and observed that both groups had comparable skills, but the group with spaced-out lessons for one month performed significantly better in microsurgical suturing.

Regarding skill retention based on the distribution of teaching over time, one study analyzed suture accuracy one month after an 8-hour course. One group completed all 8 hours in a single day, while the other group had four sessions of 2 hours each. At the end of the course, both groups had comparable skills. However, after one month, the group with spaced-out lessons performed significantly better in microsurgical suturing^30^.

**Figure 6:**
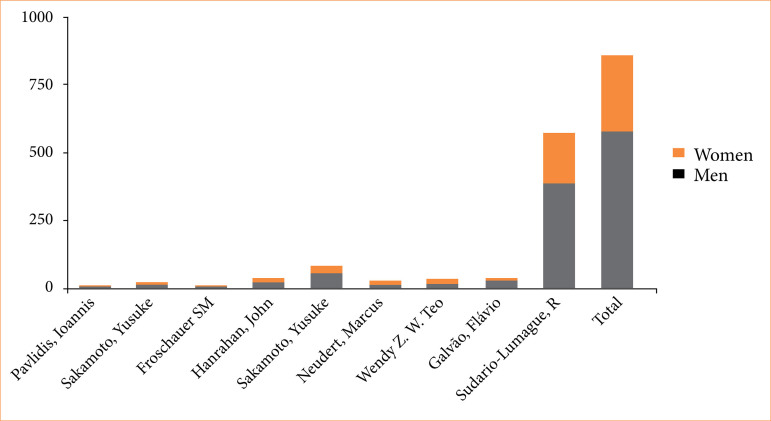
Gender stratification among the students participating in each article and among all articles in this research.

Source: Elaborated by the authors.

The average age of participants in six articles was 23.7 years. Gender stratification was cited in nine articles, 67.5% of the participants were men and 32.5% were women (Fig. 6). Sudario-Lumague et al.^27^ compared microsurgical performance between genders and demonstrated no significant difference.

Two articles^21,40^ concluded that younger individuals acquire motor skills more easily than older individuals, what encourages early teaching.

Al Omran et al.^26^ compared the effects of regular physical activity on microsurgical performance, finding that high levels of physical activity in medical students are associated with slower anastomosis and an increased number of movements for the same task. However, this does not apply to specialists. Zyluk et al.^41^ found a positive effect of caffeine and a negative effect of physical exercise on microsurgical performance shortly before the task. A small dose of alcohol taken before the task showed little performance effect.

Hanrahan et al.^28^ evaluated the role of hand tremors and anxiety in students’ microsurgical skills and observed that overall performance decreased with a higher subjective perception of anxiety. However, greater physiological tremor and higher anxiety in objective tests were not associated with a decline in microsurgical performance.

Micko et al.^42^ studied the effects of sleep deprivation in medical students and neurosurgery residents to simulate a night shift. The results showed that performance scores significantly increased and time remained stable in both groups, but both groups had worse performance.

Pavlidis et al.^22^ affirmed that manual skills are facilitated by the absence of strong sympathetic stimuli, through the elimination of potential stressors in the context of informal education. High levels of stress during surgical training trigger fight-or-flight responses and high error rates. This article, as well as the one by Fulton et al.^43^, concluded that the early introduction of microsurgery during medical school, which has a lighter routine compared to residency, reduces exogenous stress and improves skills.

Regarding the method for evaluating students’ performance, time measurements^25^ were used. A reproducible scale was employed in the article by Sudario-Lumague et al.^27^. The Stanford Microsurgery and Resident Training (SMaRT) scale was used in one article^25^. SmaRT is an established and well-known scale with nine evaluation categories, each rated from 1 to 5, resulting in a score ranging from 9 to 45. The Nagoya University Microvascular Anastomosis Assessment System was also used in one article^24^.

The global rating scale, objective structured assessment of technical skill (OSATS), University of Bergen assessment tool^31^, McGill global rating scale^44^, and direct observation of procedural skills^28^ appeared in one article each, except for the OSATS scale, which was cited in two articles. All of these are tools used to measure performance in practical and technical skills, being more comprehensive and not specific to microsurgery.

The Swedish occupational fatigue inventory^32^ evaluates work-related fatigue. The surgical task load index^32^ measures perceived workload during surgical procedures, an adaptation of the NASA task load index^22^. State trait anxiety inventory^22^ and workload in traffic control systems^28^ were utilized to assess anxiety.

Microsurgery teaching was directly linked to medical specialties and influenced students’ ambitions toward surgery as a future career^13,29,31^. In four articles^12,14–16^, the course offering was related to ophthalmology; in three^44–46^, to otorhinolaryngology; and in one^37^, to plastic surgery. These activities highlight young talent and sparks interest in microsurgery and the specialty.

Microsurgical performance comparisons between students and more experienced groups such as residents and surgeons conclude that students’ results are very similar to those of more experienced individuals, sometimes even better^13,24,29,36,47,48^.

## Discussion

The principles of microsurgical teaching, according to Mikó et al.^11^, were: activity, synchronization, video assistance, self-control, individualization, and analysis. Also, three areas of focus have been mentioned: safety training, risk management, and technical guidance. These areas are especially important when handling animals, using sharp materials and contributing to translational research^35^. To meet these principles, animals are not necessary at the initial microsurgical teaching and should be offered later if there is a genuine desire for specialization in microsurgery. This approach reduces animal suffering, respects bioethical considerations, and lessens the stress on students of handling live animals^21–23,37^. Inert models bear little resemblance to clinical situations, so they should be used as an introduction to microsurgical training with animals^38^.

The ethylene vinyl acetate model is practical, reproducible, portable, extremely cheap, readily available and allows teaching both two and three-dimensional suturing techniques^17^. The chicken thigh or wing model allows handling nerves, veins and arteries, in addition to enabling more complex procedures. However, chicken thigh and wing models’ limitations include absence of blood flow and inability to replicate thrombotic phenomena^38^. The technique taught should not be too complex in order to maintain a balance between students’ difficulty and level of instruction, ensuring lessons without significant frustration^34^.

The content regarding microsurgery for medical students is heterogeneous in purpose, time, teaching method, teacher-to-student ratio, and investment. Despite a reasonable number of reports, they are limited to a few developed countries and research centers of excellence, such as Germany, France, England, Japan, the United States, Canada, Brazil, Romania, and Hungary.

Practical learning with mentor feedback is superior to self-directed theoretical learning through instructional videos^49^. Teaching groups should be small, so each student can be closely monitored, with their mistakes corrected moment by moment, preventing the acquisition of poor manual habits. The course should be short, allowing intensive training, but spread over more than one session, so that each session is not exhausting, ensuring maximum learning with better retention.

We believe that learning this skill is feasible during medical school, where there is greater ease and availability of time to acquire technical skills. Thus, when students begin training during medical school, they gain the potential to achieve superior skills during residency practice^33^. Another reason for teaching microsurgery for medical students is their involvement in surgical research, which is valuable for research method^35^.

Finally, anxiety is an endogenous stress factor for nowadays’ medical students. The self-perception of anxiety undermines the student’s self-confidence and is even more harmful than its somatic effects, such as the intensification of physiological tremors. Thus, maximizing learning should be achieved through the reduction of stress factors.

In our Laboratory of Universidade de São Paulo Medical School, inspired by the legendary Professor Robert Zhen Zhong (*in memoriam*), we involve medical students in our research program of scientific initiation using rats as microsurgical models. For bioethical reasons, before performing surgery in animals, the students perform courses for animal use and microsurgery training using the latex glove simulator, in two weekly sessions for four weeks, with peer-assisted learning. In these sessions, the students are required to perform three continuous sutures with the extension of 3 cm on the upper face of the glove during 1 hour. After that training and other specific education, the students may participate in our experiments involving innovative translational microsurgical animal research that include intestinal transplantation^34^, Cuff-Glue sutureless anastomosis^10^, multivisceral transplantation^50,51^, anorectal transplantation^52^, hepatic ischemia-reperfusion experiments^53^, among others.

## Conclusion

Microsurgical education for medical students is possible, feasible, and expandable. However, much still needs to be researched and developed, especially regarding the expansion of this knowledge to other countries and medical reference centers.

## Data Availability

All dataset were generated or analyzed in the current study.
